# The relationship between NLR/PLR/LMR levels and survival prognosis in patients with non-small cell lung carcinoma treated with immune checkpoint inhibitors

**DOI:** 10.1097/MD.0000000000028617

**Published:** 2022-01-21

**Authors:** Na Liu, Jinmei Mao, Peizhi Tao, Hao Chi, Wenhui Jia, Chunling Dong

**Affiliations:** Department of Pulmonary and Critical Care Medicine, Second Hospital, Jilin University, Changchun, Jilin, China.

**Keywords:** immune checkpoint inhibitors, lymphocyte to monocyte ratio, neutrophil to lymphocyte ratio, non-small cell lung carcinoma, platelet to lymphocyte ratio

## Abstract

**Background:**

The relationship between neutrophil to lymphocyte ratio (NLR), platelet to lymphocyte ratio (PLR), and lymphocyte to monocyte ratio (LMR) and the dire prognosis of non-small cell lung carcinoma patients who received immune checkpoint inhibitors (ICIs) are not known yet.

**Methods:**

We screened the articles that meet the criteria from the database. The relationship between NLR/PLR/LMR levels and the survival and prognosis of non-small cell lung cancer patients treated with ICIs was analyzed. Summarize hazard ratio (HR) with 95% confidence interval (CI) to study progression-free survival (PFS) and overall survival (OS).

**Results:**

Thirty-four studies involving 3124 patients were enrolled in the final analysis. In short, high pre-treatment NLR was related to poor OS (HR = 2.13, 95% CI:1.74–2.61, *P* < .001, I^2^ = 83.3%, *P* < .001) and PFS (HR = 1.77, 95% CI:1.44–2.17, *P* < .001, I^2^ = 79.5%, *P* < .001). Simultaneously, high pre-treatment PLR was related to poor OS (HR = 1.49, 95% CI:1.17–1.91, *P* < .001, I^2^ = 57.6%, *P* = .003) and PFS (HR = 1.62, 95% CI:1.38–1.89, *P* < .001, I^2^ = 47.1%, *P* = .036). In all subgroup analysis, most subgroups showed that low LMR was related to poor OS (HR = 0.45, 95% CI: 0.34–0.59, *P* < .001) and PFS (HR = 0.60, 95% CI: 0.47–0.77, P < 0.001, I^2^ = 0.0%, *P* < .001).

**Conclusion:**

High pre-treatment NLR and pre-treatment PLR in non-small cell lung carcinoma patients treated with ICIs are associated with low survival rates. Low pre-treatment and post-treatment LMR are also related to unsatisfactory survival outcomes. However, the significance of post-treatment NLR and post-treatment PLR deserve further prospective research to prove.

## 1 Introduction

Lung carcinoma is a chief reason for worldwide morbidity and mortality, with approximately 2.094 billion cases and 1.8 million patients dying of the disease occurring annually.^[1]^ Lung cancer may be classified into 2 major groups: small cell lung cancer and non-small cell lung cancer (NSCLC) according to histopathological diagnosis.^[2,3]^ NSCLC represents about 85% of all lung carcinomas, and its incidence is rising globally. It comprises 2 predominant histological subtypes: adenocarcinoma (approximately 40%–50% cases) squamous cell carcinoma (SCC, about 20%–30% cases).^[2,4]^ Unfortunately, it is usually diagnosed in the late stages of the disease, and it is not easy for us to treat.^[5]^ Although we are not helpless in the treatment of NSCLC, and traditional therapies are also developing rapidly, the prognosis of NSCLC is still inferior. Recently, immune checkpoint inhibitors (ICIs) are aimed at cytotoxic T lymphocyte antigen 4 (CTLA-4), programmed cell death protein 1 (PD-1), or programmed cell death protein ligand 1 (PD-L1) has brought great hope for the treatment of lung carcinoma. The successful application of reagent development in various advanced carcinomas has further enriched the treatment methods of lung carcinoma.^[6–8]^

The ICIs have manifested positive results in the field of advanced carcinoma treatment. Unlike traditional chemotherapy, radiation therapy, or targeted therapy, ICIs directly restore the weak host antitumor immune response mediated by the tumor. Many advanced carcinomas had successfully used the ICIs targeting CTLA-4, PD-1, or PD-L1. So the development of ICIs has attracted significant interest from experts in tumor immunology.^[6–8]^ Although the first immune checkpoint molecule identified in 1987 was CTLA-4,^[9]^ the PD-1/PD-L1 axis has been extensively studied due to its role in CD8+ T cell failure.^[10]^ This concept was extended by tumor immunologists to the field of antitumor immunity so that PD-1/PD-L1 became one of the most prospective targets for medicine development,^[11]^ therapeutic monoclonal antibodies targeting PD-1 or PD-1L for the treatment of various advanced carcinomas have shown significant clinical efficacy.^[11,12]^ As early as 2017, the US Food and Drug Administration (FDA) has approved 5 monoclonal antibodies against PD-1 or PD-L1 to treat diverse advanced carcinomas.^[13]^ Two antibodies against PD-1, nivolumab, and pembrolizumab, and 2 antibodies against PD-L1, atezolizumab, and durvalumab, have been approved by the European Medicines Agency and the US FDA for treating advanced NSCLC.^[14–19]^ Immune checkpoint therapy was initially used as a second-line treatment, but now it has almost become an alternative to carcinoma treatment.^[17,20]^ The molecular characteristics and immune status of advanced NSCLC help determine individualized treatment options. For example, patients with epidermal growth factor receptor (EGFR) mutations, anaplastic lymphoma kinase (ALK) rearrangements, ROS1 rearrangements, BRAF mutations, NTRK mutations, and high PD-L1 levels should use FDA-approved targeted therapy or immunotherapy first-line treatment.^[21]^ In the past decade, the FDA has approved various targeted drugs for patients with operable mutations, such as EGFR, ALK, ROS1, BRAF, and NTRK.^[22]^ Promising targeted drugs for KRAS G12C, RET, MET, and AXL, and other mutations are being studied and may be approved shortly. For patients without operable mutations, if PD-L1 expression exceeds 50%, the best option is immunotherapy alone, and if PD-L1 expression is low, combined chemotherapy. The exploration of immunotherapy for advanced/metastatic NSCLC has surpassed the anti-PD-1/PD-L1 and CTLA pathways.^[21]^

Previous studies have manifested that systemic inflammation is connected with the prognosis of solid tumors.^[23]^ The ratio of neutrophils to lymphocytes (NLR) is defined as the absolute neutrophil count in the whole blood divided by the total lymphocyte count in the whole blood, which routine blood tests can quickly check, either obtain data cheaply. This value is correlated with the prognosis of many carcinomas, including lung carcinoma.^[24]^ Recently, it has been shown that NLR, platelet to lymphocyte ratio (PLR), and lymphocyte to monocyte ratio (LMR) are markers of systemic inflammation related to the prognosis of various carcinomas.^[25–31]^ Although there is a meta-analysis to study the prognostic value of NLR and PLR in NSCLC patients who received ICIs, they only focus on the 2 indicators of NLR and PLR.^[32]^ However, the correlation between LMR and survival prognosis in NSCLC patients treated with ICIs is unknown. Moreover, as new research continues to be reported, it's worth updating this meta-analysis to explore the prognostic effect of LMR in NSCLC patients who received ICIs and further clear and definite the correlation between NLR and PLR and the poor prognosis of NSCLC patients treated with ICIs.

## 2 Material and method

### 2.1 Ethical statement

Since this meta-analysis is based on published data and does not involve patient recruitment and personal information collection protocols, it does not require the ethics committee's approval.

### 2.2 Literature search

This meta-analysis follows the guidelines from the preferred reporting items for systematic reviews and meta-analyses checklist.^[33]^

As of November 30, 2020, a comprehensive literature search has been conducted in PubMed, EMBASE, Cochrane Library, and Web of Science databases. The specific search strategy is shown in A1, Supplemental Digital Content.

Registered with PROSPERO before writing this article (http://www.crd.york.ac.uk/prospero/, number: CRD42021219001).

### 2.3 Inclusion and exclusion criteria

Included articles: immunotherapy of advanced NSCLC patients treated with ICIs; analysis of the relationship between poor prognostic and pretreatment and/or posttreatment NLR/PLR/LMR; provide a hazard ratio (HR) of 95% confidence interval (CI) for progression-free survival (PFS) and/or overall survival (OS) according to NLR/PLR/LMR; and full text is available.

Excluded articles: repeated research; reviews, letters, case reports, or nonrelevant studies; not written in English; and not enough data available.

### 2.4 Data extraction and quality assessment

Two participants searched whole the articles that needed to be extracted. They extracted the first author's name, publication year, region, and the number of patients included, follow-up time, treatment strategy, survival result, HR with 95% CI, NLR/PLR/LMR cutoff value, and test time. Disagreements were resolved through discussions between 2 investigators.

The Newcastle–Ottawa Scale evaluated articles that meet the inclusion criteria. If the evaluation score of this article was ≥5, it was considered high quality (A2, Supplemental Digital Content).

### 2.5 Data synthesis and analysis

To study the relationship between pretreatment/post-treatment NLR/PLR/LMR and the survival outcome of NSCLC patients receiving ICIs, HRs and 95% CI were directly extracted from eligible studies, and HRs with 95% CI were combined to obtain valid value. PFS or OS is mainly used to assess prognostic results. The heterogeneity of the summary results is evaluated by Cochran Q test and I^2^ statistics. When the *P* value of the Cochran Q test is ≤0.05 and/or the I^2^ value ≥50%, it is considered to be significant heterogeneity. All random effects models will be used regardless of the heterogeneity, considering the statistical heterogeneity. Subgroup analysis was performed based on cutoff value, sample size, area, test time point, and follow-up time, and sensitivity analysis was used to explore the heterogeneity of research results further. Using a funnel chart, Egger test assesses publication bias. All calculations are performed by Stata 14.0 (StataCorporation, CollegeStation, TX).

## 3 Results

### 3.1 Literature search and study characteristic

**Figure 1:**
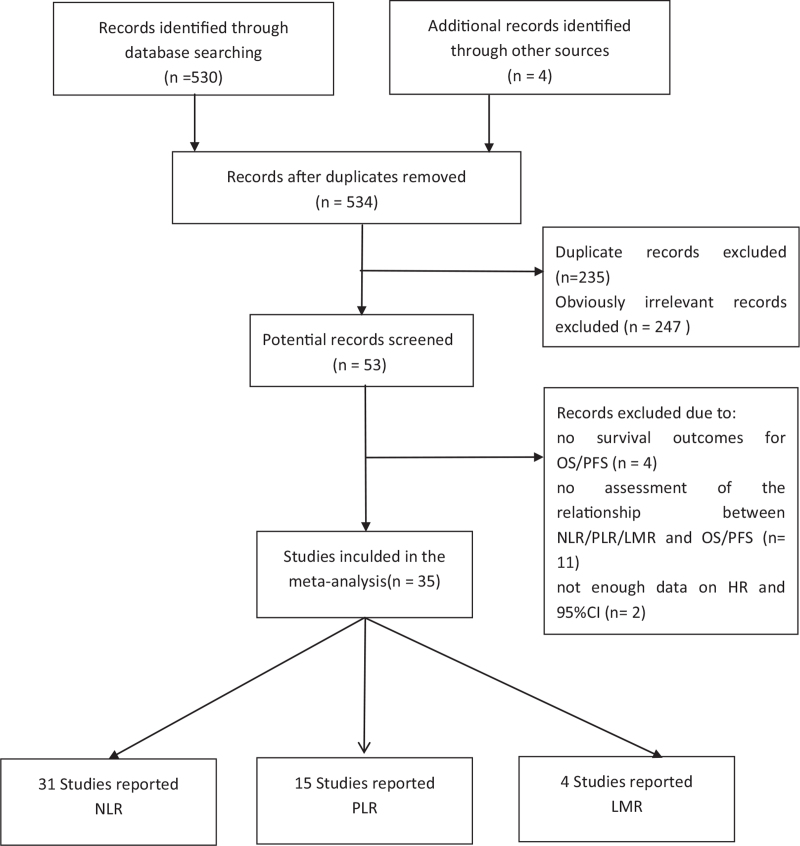
Flow diagram of the studies selection procedure. A total of 534 articles were retrieved initially. After careful review, 500 articles were excluded due to various reasons. Finally, the analysis included 31 studies reporting the NLR, 15 studies reporting the PLR, and 4 studies reporting the LMR. CI = confidence interval, HR = hazard ratio, LMR = lymphocyte-to-monocyte ratio, NLR = neutrophil–lymphocyte ratio, OS = overall survival rate, PFS = progression-free survival, PLR = platelet–lymphocyte ratio.

Five hundred thirty-four articles were obtained initially. Two hundred thirty-five articles were deleted through the duplicate check. After preliminary screening of the headline and abstract, weeded out 247 studies based on the inclusion and exclusion criteria mentioned in the previous summary. Afterward, we conducted a more in-depth review of the full text of the remaining 53 articles. Figure 1 shows in detail the flow chart of authors screening articles. Finally, 19 studies met the inclusion criteria: 5 studies didn’t provide meaningful results, 11 didn’t provide cutoff values for NLR/PLR/LMR, 3 did not provide sufficient HR and 95% CI, and 1 overlapped data research. Finally, a total of 34 studies^[34–67]^ were included, of which 31 studies reported NLR^[34–39,47–53,55–67]^ and 15 studies reported PLR,^[35,40,43,44,47,49–51,54–56,59,62,65,66]^ 4 studies reported LMR.^[49,55,60,61]^ The features of the included researches are demonstrated in Table 1.

**Table 1:** The features of the included researches.

	Cutoff											
Author	NLR	PLR	LMR	Outcomes	Year	Follow-up (months)	Time point	Region	Study design	Sample size	Age	Treatment
Bagley et al	5			OS/PFS	2017	NR	Pre	America	R	175	68 (33–88)	Nivo
Diem et al	5	262		OS/PFS	2017	NR	Pre	Europe	R	52	66 (46–88)	Nivo
Patil et al	2.8			OS	2017	NR	Pre	America	P	115	67 (45–90)	Nivo
Rogado et al	5			OS/PFS	2017	NR	Pre	Europe	R	40	67	Nivo
Soyano et al	4.59			OS/PFS	2017	NR	Pre	America	R	52	NR	Nivo, Pemb
Alone	4			OS	2018	5.3	Pre	Europe	R	88	64 (31–81)	Nivo, Pemb
Fukui et al	5			OS	2018	10.9	Pre	Japan	P	52	69 (46–83)	Nivo
Facchinetti et al	4			OS	2018	12.6	Pre	Italy	P	54	69 (43–85)	Nivo
Park et al	5			PFS	2018	11.5	Pre and Post	America	R	159	68 (41–91)	Nivo
Russo et al		160		OS/PFS	2018	17	Pre	Italy	R	28	69 (47–78)	Nivo
Shiroyama et al	4			PFS	2018	12.4	Pre	Japan	R	201	68 (27–87)	Nivo
Suh et al	5	169		OS/PFS	2018	26.2	Post	Korea	R	54	68 (43–80)	Nivo, Pemb
Takeda et al	5	150		PFS	2018	NR	Pre and Post	Japan	R	30	71 (54–83)	Nivo
Amaral et al				OS/PFS	2019	NR	Pre	Europe	R	32	61 (40–82)	Nivo, Pemb
Dusselier et al	5	262		OS	2019	NR	Pre and Post	Europe	R	59	59 (30–87)	Nivo
Katayama et al	5	262	1.7	OS/PFS	2019	NR	Pre	Japan	R	35	70 (40-83)	Nivo, Pemb, Atez
Liu et al	3.07	144		OS/PFS	2019	6.9	Pre	China	R	44	60 (43–74)	Nivo
Miriam	5.2			OS	2019	9.7	Pre	Germany	P	35	65 (24–85)	Nivo, Pemb
Prelaj et al	4			PFS	2019	NR	Pre	Italy	R	193	65 (30–88)	PD-1/PDL-1
Pavan et al	3	180		OS/PFS	2019	56.3	Pre	Italy	R	174	67.3 (37–83)	Nivo, Pemb, Atez
Ren et al	2.5			OS/PFS	2019	31.2	Pre	China	R	147	57.6	Nivo, Pemb
Jiang et al		168.13		OS/PFS	2020	7.1	Pre	China	R	76	61 (35–74)	Nivo, Durv
Katayama et al	5	262	1.5	OS/PFS	2020	NR	Pre	Japan	R	81	71 (42–84)	Atez
Matsubara et al	5	150		OS	2020	NR	Pre	Japan	R	24	64.5 (49–82)	Atez
Prelaj et al	4		1.8	OS/PFS	2020	NR	Pre and Post	Italy	R	154	67 (31–86)	Nivo, Pemb
Petrova et al	5	200		OS/PFS	2020	NR	Pre	Bulgaria	R	119	62.3 (54.4–70.2)	Pemb
Peng et al	5			OS/PFS	2020	NR	Pre	China	R	102	62	PD-1/PDL-1
Rossi et al	4.9		1.38	OS	2020	NR	Pre and Post	Italy	R	65	68 (39–86)	Nivo
Russo et al	5	200		OS	2020	NR	Pre	Italy	R	187	67 (34–83)	Nivo
Simonaggio et al	3.4			OS/PFS	2020	16.8	Pre	French	R	75	65 (31.2–86.7)	Nivo
Song et al	4			PFS	2020	NR	Pre	China	P	63	61 (39–81)	PD-1/PDL-1
Takada et al	6.05	245		OS/PFS	2020	NR	Pre	Japan	R	226	66 (31–88)	Nivo, Pemb
Xiong et al	5	169		PFS	2020	NR	Pre and Post	China	R	41	61 (42–80)	PD-1/PDL-1
Yuan et al	3.9			OS/PFS	2020	NR	Pre	China	R	92	64.5 (55.3–70.0)	PD-1/PDL-1

### 3.2 The effect of NLR on OS and PFS

**Figure 2:**
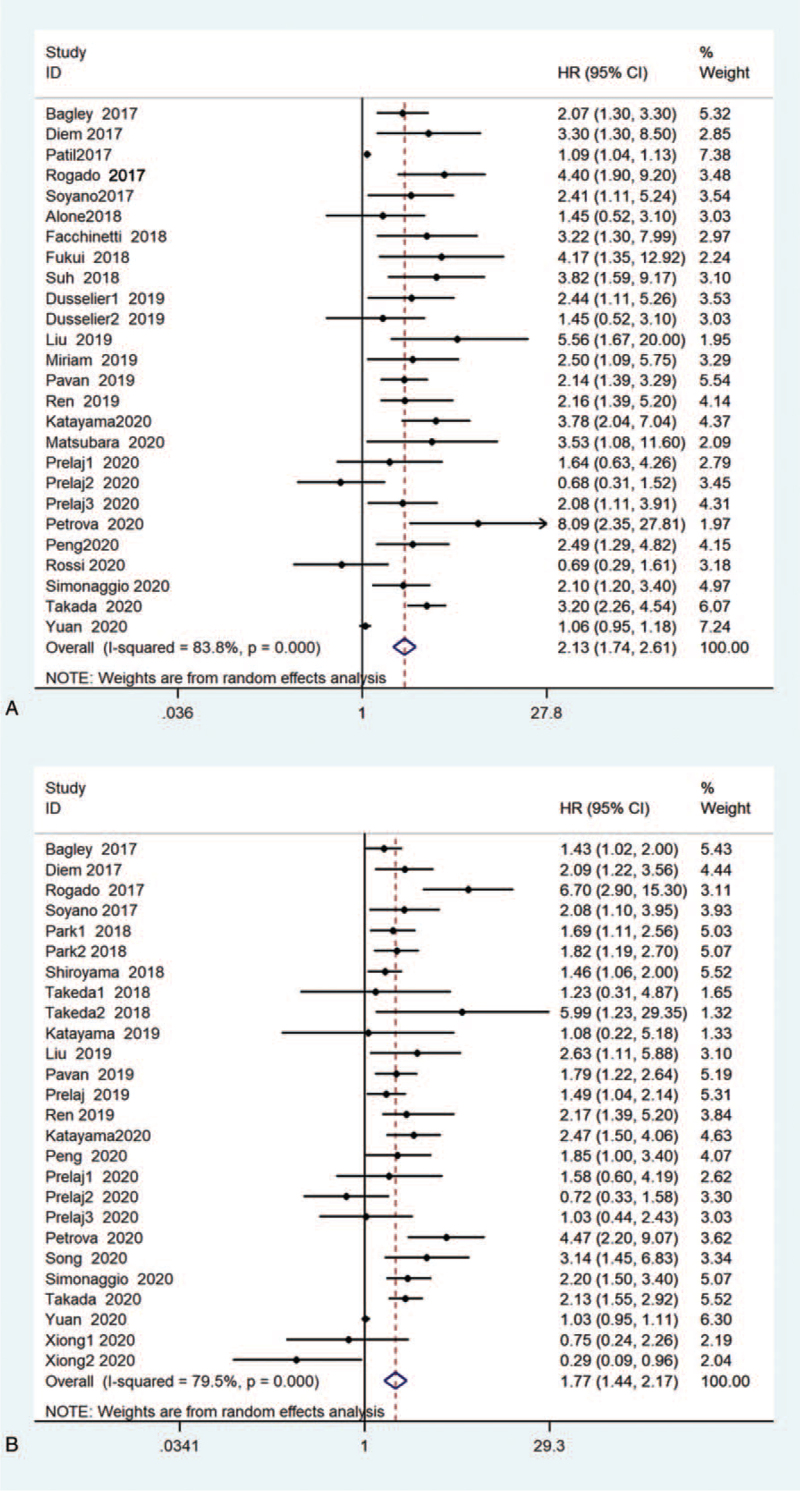
Forest plot of the relationship between NLR and survival outcomes in NSCLC patients treated with ICIs. (A) Use a random-effects model to evaluate the impact of NLR on OS. The summary results showed that high NLR was significantly associated with poor OS; (B) Random-effects model was used to evaluate the impact of NLR on PFS. The combined results showed that increased NLR was associated with poor PFS. CI = confidence interval, HR = hazard ratio, ICIs = immune checkpoint inhibitors, NLR = neutrophil–lymphocyte ratio, NSCLC = nonsmall cell lung cancer, OS = overall survival rate, PFS = progression-free survival.

In the aggregate, 31 studies indicated the correlation between NLR levels and survival endings in NSCLC sufferers accepting ICIs (Fig. 2). Throughout all researches, 26 estimated OS.^[34–38,42,43,45,55–65,67]^ Three studies have demonstrated the prognostic value of NLR in OS before and after treatment.^[44,47,60]^ The aggregated results showed that patients with high NLR had worse OS than patients with low NLR (HR = 2.13, 95% CI:1.74–2.61, *P* < .001). Significant heterogeneity was found between studies (I^2^ = 83.8%, *P* < .001). 28 researches estimated the correlation among NLR levels and PFS.^[34,35,37,39,41–44,49–53,55,58–60,63–67]^ Only 4 studies have estimated the prognostic value of NLR on PFS before and after immunotherapy.^[39,44,60,66]^ Eventually, the overall outcomes displayed that increased NLR was markedly related to worse PFS (HR = 1.77, 95% CI:1.44–2.17, *P* < .001, I^2^ = 79.5%, *P* < .001).

**Figure 3:**
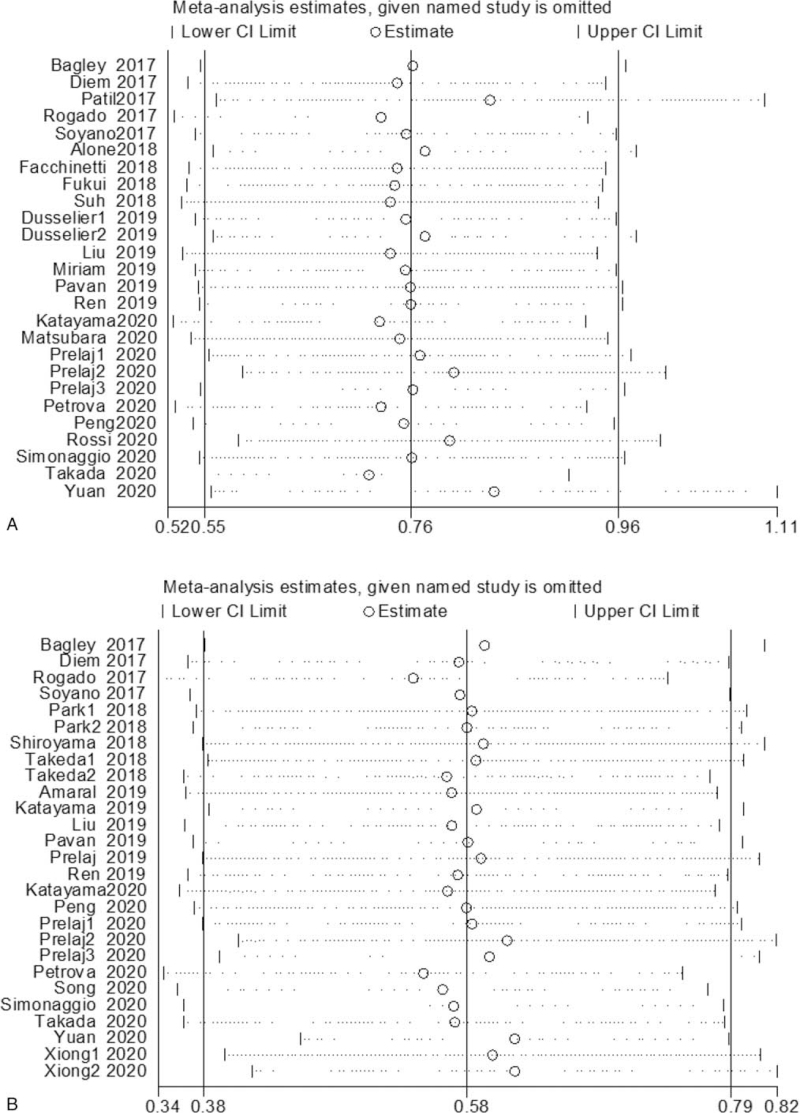
Sensitivity analysis of the relationship between NLR and survival outcomes in NSCLC patients treated with ICIs. (A) Sensitivity analysis of OS shows that Patil et al, Prelaj et al, Rossi et al, Yuan et al research is the primary source of heterogeneity; (B) sensitivity analysis of PFS shows that Rogado et al, Prelaj et al, Petrova et al, Yuan et al, Xiong et al research is the primary source of heterogeneity. CI = confidence interval, ICIs = immune checkpoint inhibitors, NLR = neutrophil–lymphocyte ratio, NSCLC = nonsmall cell lung cancer, OS = overall survival rate, PFS = progression-free survival.

Subsequently, we made further efforts to process sensitivity analysis to probe the latent heterogeneity of OS and PFS (Fig. 3). After removing the data of Patil et al,^[36]^ Prelaj et al,^[60]^ Rossi et al,^[61]^ and Yuan et al,^[67]^ the heterogeneity of OS was distinctly dropped (I^2^ = 0.0%; *P* = .605), and the combined HR was 2.61 (95% CI:2.26–3.01, *P* < .001). Perhaps this was the origin of heterogeneity. After removing 5 studies, Rogado et al,^[37]^ Prelaj et al,^[60]^ Petrova et al,^[59]^ Yuan et al,^[67]^ and Xiong et al,^[66]^ the heterogeneity of PFS was distinctly reduced (I^2^ = 0.0%). Increased NLR is related to bad OS and PFS in NSCLC sufferers who received ICIs.

### 3.3 The effect of PLR on OS and PFS

**Figure 4:**
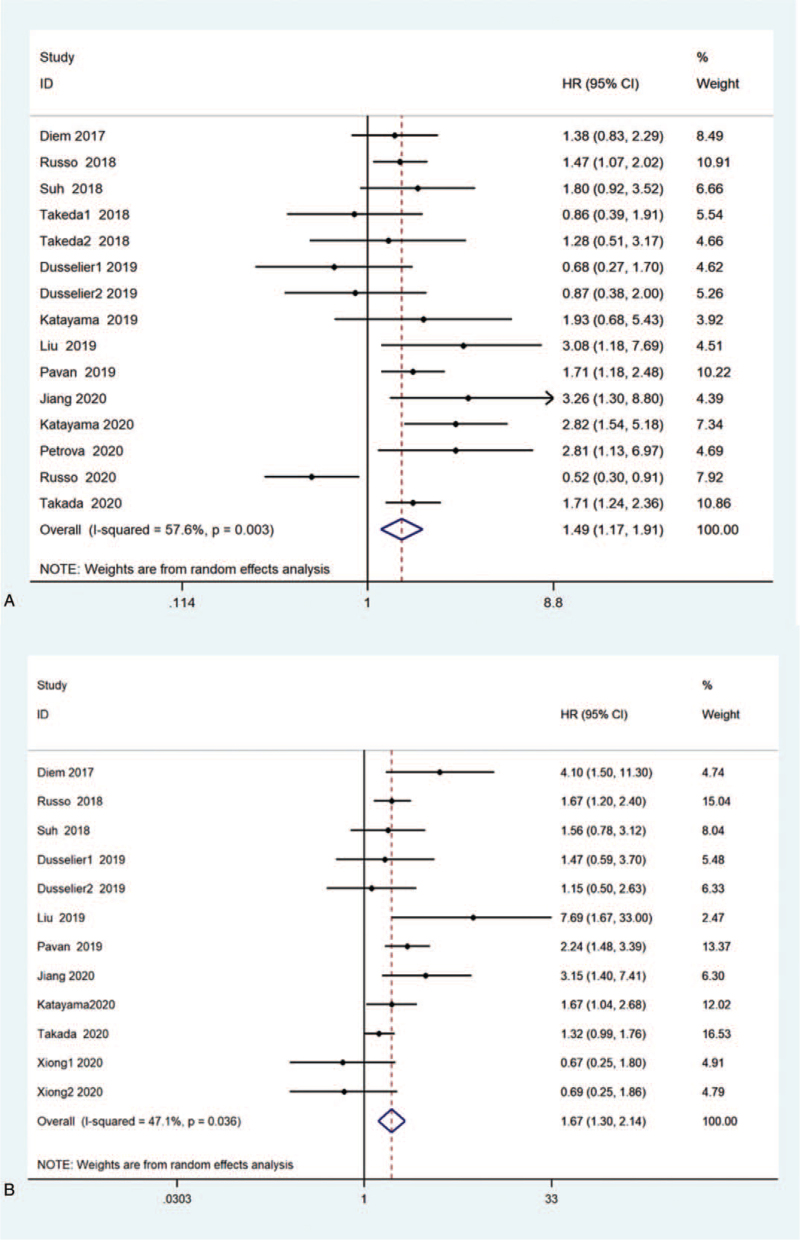
Forest plot of the relationship between PLR and survival outcome in NSCLC patients treated with ICIs. (A) Use a random-effects model to evaluate the impact of PLR on OS. The summary results show that higher PLR is associated with worse OS; (B) Random-effects model is used to evaluate the influence of PLR on PFS. The summary results show that elevated PLR is significantly associated with poor PFS. CI = confidence interval, HR = hazard ratio, ICIs = immune checkpoint inhibitor, NSCLC = nonsmall cell lung cancer, OS = overall survival rate, PFS = progression-free survival, PLR = platelet–lymphocyte ratio.

15 studies demonstrated the relationship among PLR value and OS.^[36,41,44,48,50–52,55–57,60,63,66]^ Counted HR after consolidation and its 95% CI (HR = 1.49, 95% CI: 1.17–1.91, *P* < .001, I^2^ = 57.6%, *P* = .003; Fig. 4). The gathered analysis of 12 researches also disclosed^[36,41,44,45,50–52,55,56,60,66–68]^ that high PLR was obviously related to poorer PFS (HR = 1.62, 95% CI: 1.38–1.89, *P* < .001, I^2^ = 47.1%, *P* = .036).

**Figure 5:**
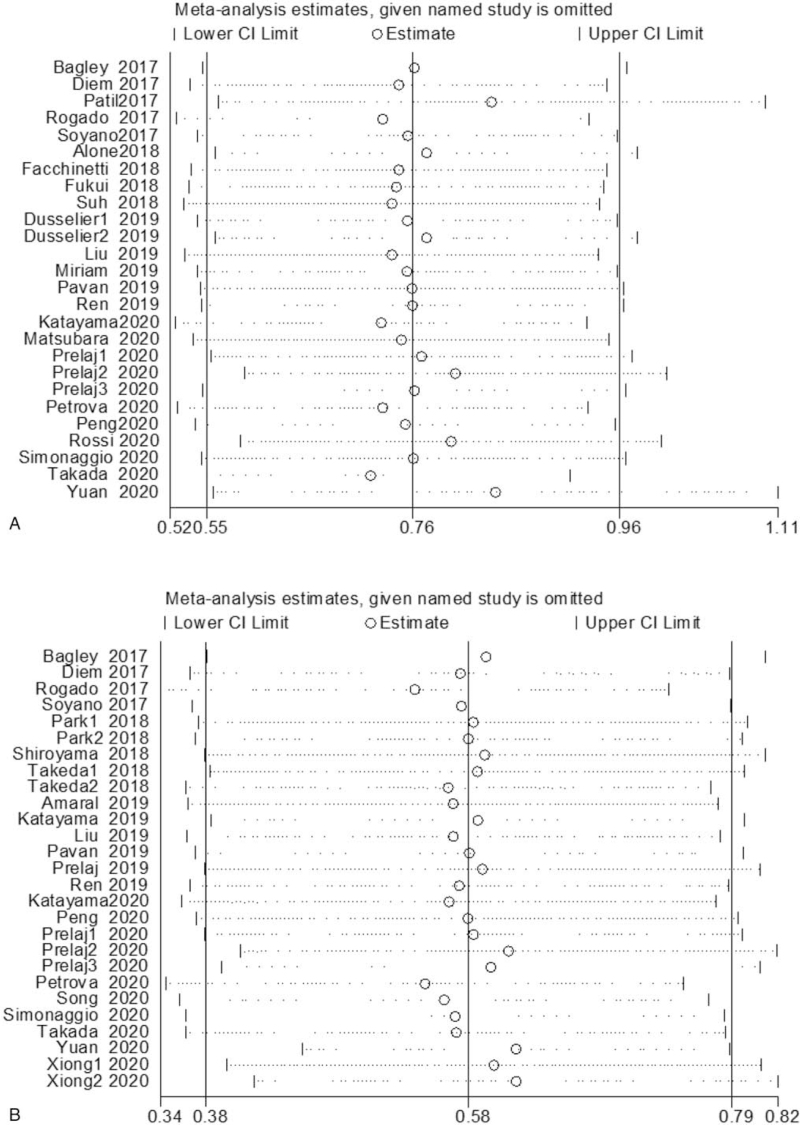
Sensitivity analysis of the relationship between PLR and survival outcomes in NSCLC patients treated with ICIs. (A) Sensitivity analysis of OS shows that the research of Russo et al, Takada et al, Dusselier et al is the primary source of heterogeneity; (B) sensitivity analysis of PFS shows that the research of Takada et al and Xiong et al is the primary source of heterogeneity. CI = confidence interval, ICIs = immune checkpoint inhibitors, NSCLC = nonsmall cell lung cancer, OS = overall survival rate, PFS = progression-free survival, PLR = the ratio of platelets to lymphocytes.

We subsequently conducted a sensitivity analysis (Fig. 5). Concerning OS, after removing the records of Russo et al,^[62]^ Takada et al,^[44]^ and Dusselier et al,^[47]^ the heterogeneity was visibly decreased (I^2^ = 0.0%; *P* = .535). As far as PFS is concerned, after eliminating the data of Takada et al^[65]^ and Xiong et al,^[66]^ the heterogeneity is decreased (I^2^ = 23.5%; *P* = .234).

### 3.4 The effect of LMR on OS and PFS

**Figure 6:**
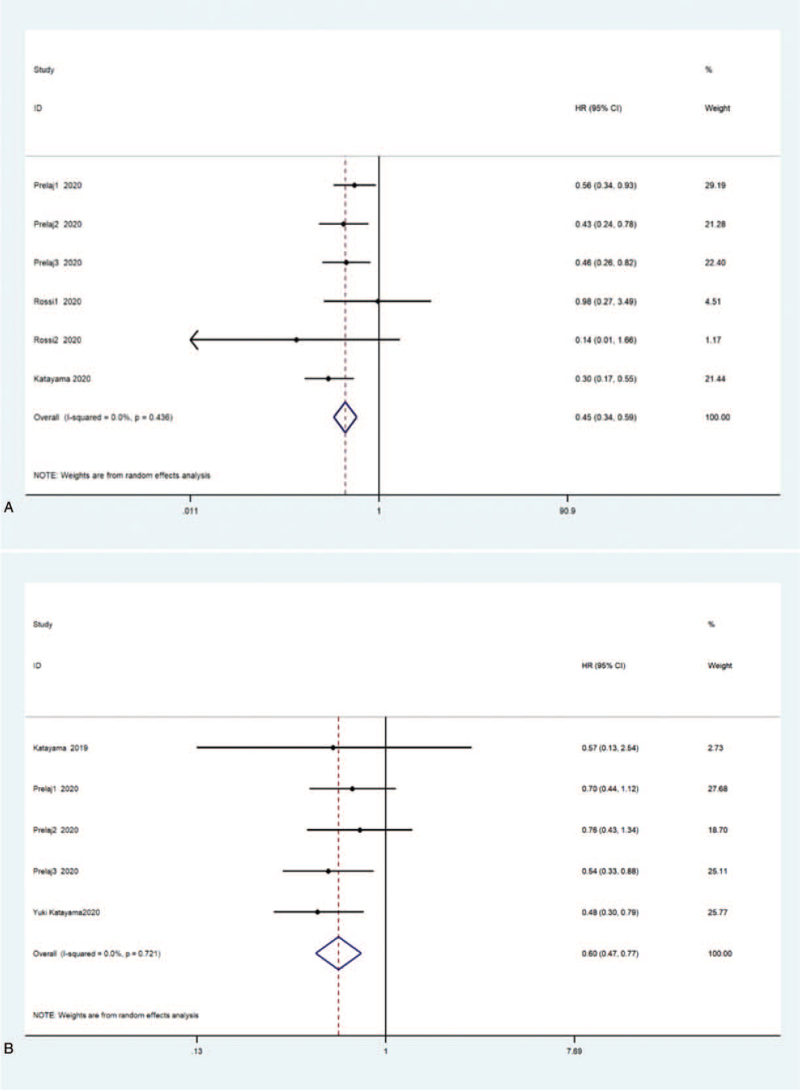
Forest plot of the relationship between LMR and survival outcome in NSCLC patients treated with ICIs. (A) Use a random-effects model to evaluate the impact of LMR on OS. The summary results show that lower LMR is associated with worse OS; (B) random-effects model is used to evaluate the impact of LMR on PFS. The summary results show that reduced LMR is significantly associated with poor PFS. CI = confidence interval, HR = hazard ratio, ICIs = immune checkpoint inhibitor, LMR = ratio of lymphocytes to monocytes, NSCLC = nonsmall cell lung cancer, OS = overall survival rate, PFS = progression-free survival.

On the whole, 4 studies have demonstrated the correlation between LMR levels and the survival prognosis of NSCLC patients who received ICIs.^[49,55,60,61]^ In these studies, 3 data evaluated OS.^[55,60,61]^ 2 studies explored the prognostic value of LMR before and after treatment of OS.^[61,62]^ The summary results showed that the OS of patients with low LMR was distinctly worse than that of patients with high LMR (HR = 0.45, 95% CI: 0.34–0.59, *P* < .001; Fig. 6). And heterogeneity wasn’t discovered among the studies (I^2^ = 0.0%, *P* < .001). Three studies assessed the relationship between LMR value and PFS.^[49,55,60]^ Only 1 research studied the prognostic value of LMR on PFS before and after immunotherapy.^[60]^ The summary results likewise demonstrated that reduced LMR was visibly associated with bad PFS (HR = 0.60, 95% CI: 0.47–0.77, *P* < .001, I^2^ = 0.0%, *P* < .001).

### 3.5 Subgroup analysis

If stratified according to the cutoff value, sample size, region, test time point, and follow-up time, the heterogeneity between the various data will be better revealed. Table 2 summarizes the results of all subgroup analyses.

**Table 2:** Results of subgroup analysis.

		OS					PFS			
		Association	Heterogeneity				Association	Heterogeneity		
Anlalysis	N	HR (95%) CI	*P*	I^2^	*P*	N	HR (95%) CI	*P*	I^2^	*P*
**NLR**										
Total	26	2.13 (1.74,2.61)	<.001	83.80%	<.001	26	1.77 (1.44,2.17)	<.001	79.50%	<.001
Subgroup analysis cutoff value										
≥5	12	2.92 (2.41,3.54)	<.001	0.00%	.519	14	1.94 (1.48,2.55)	<.001	62.20%	.001
<5	14	1.53 (1.26,1.85)	<.001	73.10%	0	12	1.61 (1.24,2.07)	<.001	76.30%	0
Sample size										
>60	16	1.78 (1.43,2.23)	<.001	86.00%	0	17	0.55 (0.33,0.76)	<.001	81.40%	0
≤60	11	3.0 (2.29,3.95)	<.001	0.00%	.822	10	0.65 (0.13,1.17)	.014	64.30%	.003
Region										
Asia	9	2.84 (1.66,4.86)	<.001	89.30%	0	13	1.65 (1.20,2.28)	.002	80.10%	0
America	4	2.05 (1.09,3.86)	.026	87.00%	0	4	1.65 (1.34,2.04)	<.001	0.00%	.698
Europe	13	1.92 (1.44,2.56)	<.001	44.90%	.04	10	2.01 (1.45,2.79)	<.001	65%	.02
Follow-up period (mo)										
>12	5	2.33 (1.78,3.05)	<.001	0.00%	.724	4	1.77 (1.44,2.16)	<.001	0.00%	.412
≤12	4	2.71 (1.55,4.74)	<.001	20.20%	.289	3	1.83 (1.39,2.42)	<.001	0.00%	.648
NR	17	1.94 (1.54,2.44)	<.001	85.40%	0	20	1.75 (1.33,2.29)	<.001	80.70%	0
Time point										
Pre	19	2.20 (1.75,2.76)	<.001	86.60%	0	23	1.92 (1.56,2.38)	<.001	80.50%	0
Post	7	1.90 (1.23,2.92)	.004	44.50%	.094	4	0.94 (0.37,2.37)	.9	67.90%	.025
**PLR**										
Total	15	1.49 (1.17,1.91)	.001	57.60%	.003	12	1.62 (1.38,1.89)	<.001	47.10%	.036
Subgroup analysis cutoff value										
≥200	8	1.35 (0.88,2.06)	.172	72.10%	.25	5	1.52 (1.14,2.03)	.004	21.80%	.276
<200	7	1.63 (1.29,2.07)	<.001	15.40%	.016	7	1.73 (1.16,2.58)	.007	57.60%	.028
Sample size										
>60	8	1.73 (1.08,2.78)	.023	78.20%	.253	6	1.80 (1.28,2.53)	.001	55.30%	.081
≤60	7	1.37 (1.09,1.73)	.006	9.30%	.558	6	1.54 (1.03,2.32)	.037	49.80%	.052
Region										
Asia	8	1.87 (1.43,2.46)	<.001	20.30%	.031	7	1.53 (1.04,2.25)	.032	55.20%	.037
America	0									
Europe	7	1.19 (0.83,1.73)	.346	68.00%	.153	5	1.87 (1.40,2.49)	<.001	20.60%	.283
Follow-up period (mo)										
>12	3	1.59 (1.27,2.00)	<.001	0.00%	.774	3	1.84 (1.44,2.36)	<.001	0.00%	.501
≤12	2	3.17 (1.62,6.18)	.001	0.00%	.934	2	3.94 (1.85,8.39)	<.001	4.60%	.306
NR	10	1.29 (0.89,1.85)	.175	66.00%	.207	7	1.36 (0.99,1.87)	.058	35.30%	.159
Time point										
Pre	12	1.54 (1.16,2.04)	.003	64.40%	.001	9	1.83 (1.37,2.43)	<.001	53.30%	.029
Post	3	1.33 (0.85,2.10)	.214	0.00%	.408	3	1.18 (0.74,1.89)	.484	0.00%	.422
**LMR**										
Total	6	0.45 (0.34,0.59)	<.001	0.00%	<.001	5	0.60 (0.47,0.77)	<.001	0.00%	<.001
Subgroup analysis cutoff value										
≥1.8	3	0.49 (0.35,0.67)	<.001	0.00%	.777	3	0.65 (0.49,0.87)	.004	0.00%	.626
<1.8	3	0.39 (0.16,0.96)	.04	38.70%	.196	2	0.49 (0.31,0.77)	.002	0.00%	.829
Sample size										
≥80	4	0.44 (0.33,0.58)	<.001	0.00%	.467	4	0.60 (0.47,0.77)	<.001	0.00%	.557
<80	2	0.51 (0.08,3.08)	.459	45.50%	.176	1				
Region										
Asia	1					2	0.49 (0.31,0.77)	.002	0.00%	.829
America	0									
Europe	5	0.50 (0.37,0.68)	<.001	0.00%	.631	3	0.65 (0.49,0.87)	.004	0.00%	.626
Time point										
Pre	3	0.48 (0.27,0.84)	.011	49.60%	.138	3	0.58 (0.42,0.81)	.001	0.00%	.546
Post	3	0.43 (0.29,0.69)	<.001	0.00%	.663	2	0.62 (0.43,0.91)	.013	0.00%	.372

When stratified by critical value, the study with NLR critical value ≥5 (HR of OS and PFS were 2.92 and 1.94) was more significant than that with NLR critical value <5 (HR of OS and PFS were 1.53 and 1.61, respectively). This phenomenon manifested that the patient's prognosis would worsen as the NLR levels increased. Similarly, studies with cutoff values ≥5 (I^2^ of OS and PFS = 0.0% and 62.2%) have lower heterogeneity than cutoff values of <5 (I^2^ of OS and PFS = 73.1% and 76.3%). Most subgroup analyses revealed a distinct correlation among higher NLR and worse OS and PFS, but there was no significant association among post-treatment NLR and PFS (HR = 0.94, 95% CI: 0.37–2.37, *P* = .9).

Throughout the subgroup analysis, most results revealed a distinct correlation among high PLR levels and worse survival outcomes of patients. There was no distinct association among post-treatment PLR, OS, and PFS when stratified by test time points. The combined HR were 1.33 (95% CI: 0.85–2.10, *P* = .214) and 1.18 (95% CI:0.74–1.89, *P* = .484). When stratified by country/region, there was no association among PLR and OS in the European group (HR:1.19, 95% CI: 0.83–1.73, *P* = .346). When stratified by cutoff point, there was no association among PLR and OS in the cutoff value ≥ 200 group (HR:1.35, 95% CI: 0.88–2.06, *P* = .172).

In all subgroup analyses, most subgroups revealed that low LMR was associated with bad OS (HR = 0.45, 95% CI: 0.34–0.59, *P* < .001) and PFS (HR = 0.60, 95% CI: 0.47–0.77, *P* < .001, I^2^ = 0.0%, *P* < .001). There was no correlation between LMR and OS in the sample size <80 groups (HR:0.61, 95% CI: 0.08–3.08, *P* = .459).

### 3.6 Publication bias

Publication bias is evaluated by funnel chart and Egger test. For the effects of NLR on OS and PFS, the asymmetry of the funnel chart indicates low publication bias, and the Egger test also verifies this (OS: *P*_*Egger*_ < .001, PFS: *P*_*Egger*_ < .001). As shown in Figure 7  , for the effect of PLR on OS and PFS, since the funnel chart is basically symmetric, there is no obvious publication bias in these studies. Egger test (OS: *P* = .955, PFS: *P* = .837) further proves the higher publication bias. For the effect of LMR on OS and PFS, the funnel chart is basically symmetrical, which indicates that there is no obvious publication bias in these studies. In addition, the Egger test confirms the HR for OS (*P* = .828) or PFS (*P* = .973) as a result.

**Figure 7:**
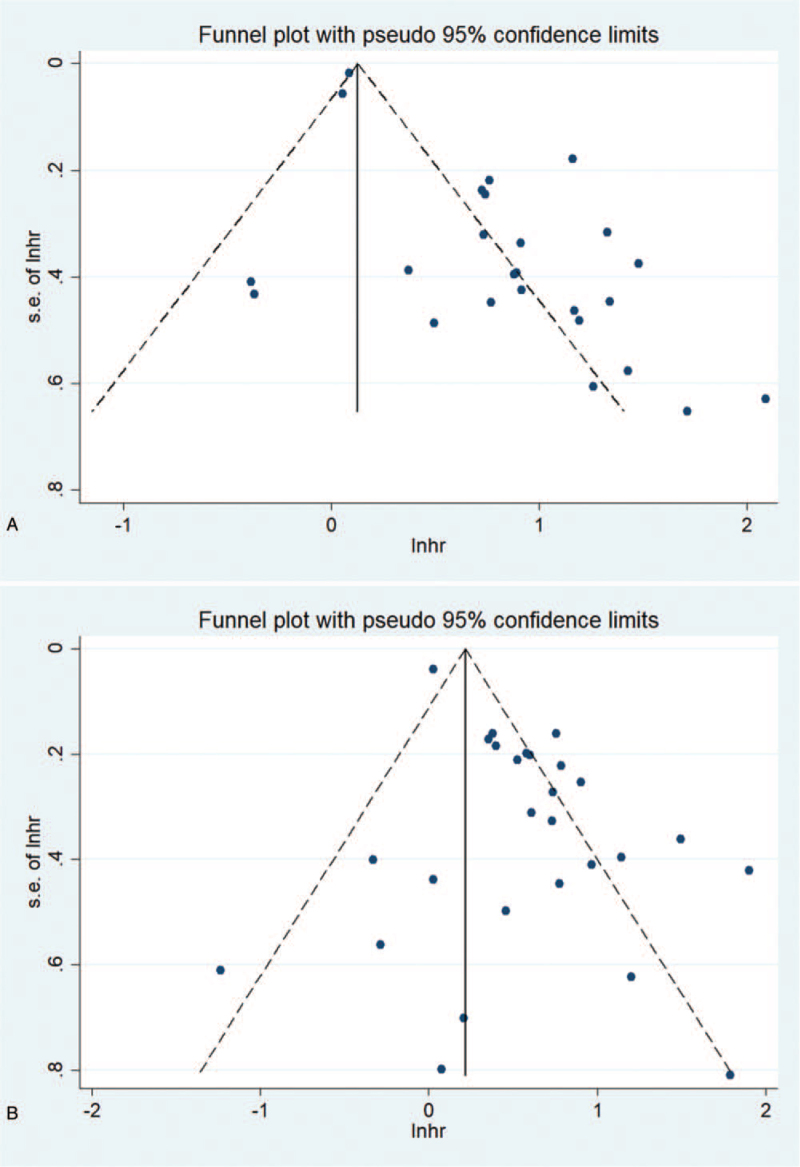
Funnel chart used to assess publication bias in survival outcomes. The funnel diagram of the association between NLR and OS (A) and PFS (B) is basically not symmetrical. The funnel diagram of the association between PLR and OS (C) and PFS (D) is basically symmetrical. The funnel diagram of the correlation between LMR and OS(E) and PFS(F) is basically symmetrical. LMR = lymphocyte to monocyte ratio, NLR = neutrophil–lymphocyte ratio, OS = overall survival rate, PFS = progression-free survival, PLR = platelet–lymphocyte ratio.

**Figure 7 (Continued):**
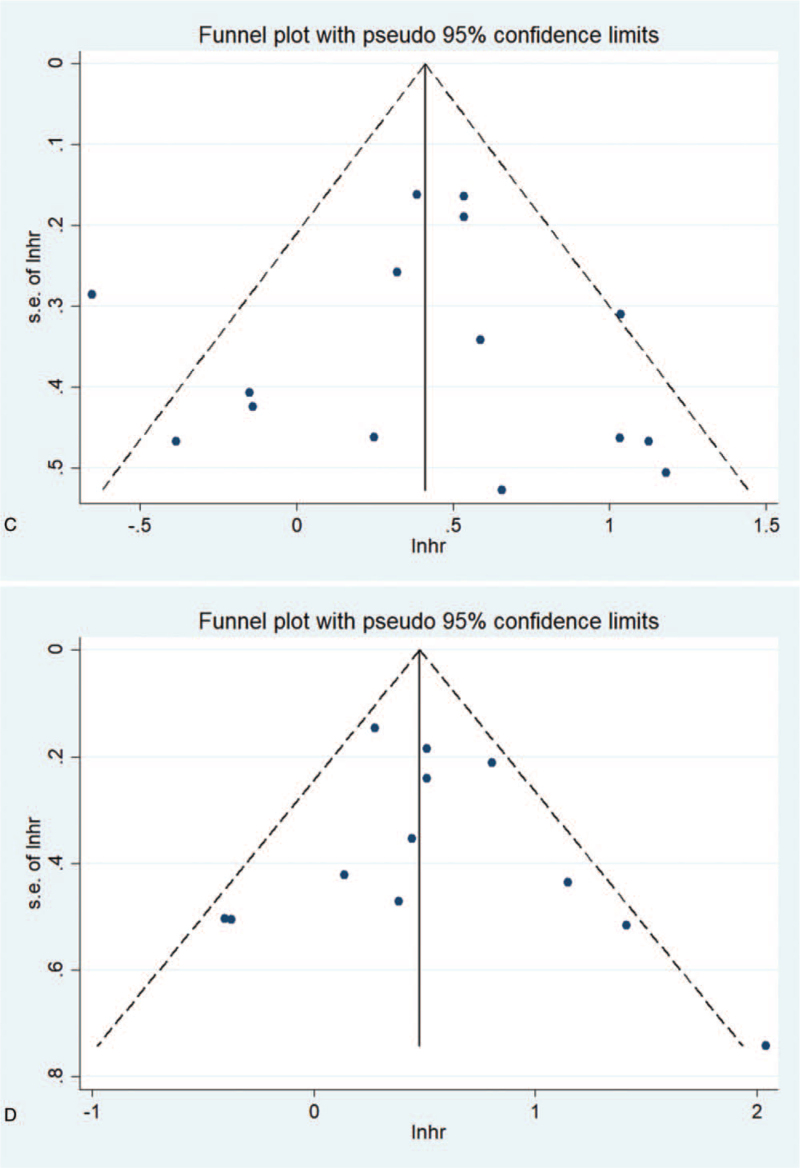
Funnel chart used to assess publication bias in survival outcomes. The funnel diagram of the association between NLR and OS (A) and PFS (B) is basically not symmetrical. The funnel diagram of the association between PLR and OS (C) and PFS (D) is basically symmetrical. The funnel diagram of the correlation between LMR and OS(E) and PFS(F) is basically symmetrical. LMR = lymphocyte to monocyte ratio, NLR = neutrophil–lymphocyte ratio, OS = overall survival rate, PFS = progression-free survival, PLR = platelet–lymphocyte ratio.

**Figure 7 (Continued):**
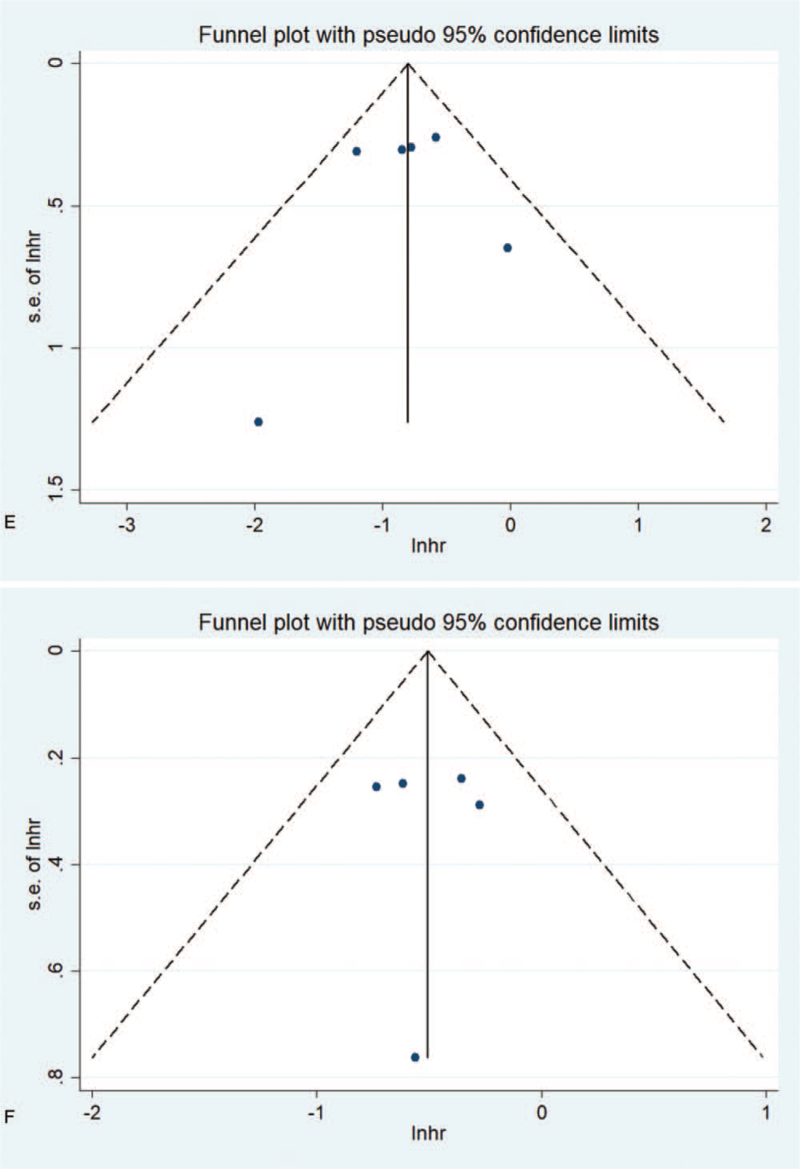
Funnel chart used to assess publication bias in survival outcomes. The funnel diagram of the association between NLR and OS (A) and PFS (B) is basically not symmetrical. The funnel diagram of the association between PLR and OS (C) and PFS (D) is basically symmetrical. The funnel diagram of the correlation between LMR and OS(E) and PFS(F) is basically symmetrical. LMR = lymphocyte to monocyte ratio, NLR = neutrophil–lymphocyte ratio, OS = overall survival rate, PFS = progression-free survival, PLR = platelet–lymphocyte ratio.

## 4 Discussion

It is universally believed that inflammation plays an essential role in tumor development and can influence the survival ending of carcinoma patients. Many studies have shown that NLR, PLR, and LMR are all associated with a bad prognosis of solid tumors.^[25,27,28,69,70]^ In our current research, we gathered 34 studies on 3124 NSCLC sufferers who accepted ICIs and thoroughly assessed the survival impact of NLR, PLR, and LMR in NSCLC patients who received ICIs treatment. We can conclude that higher NLR, higher PLR, and lower LMR are correlated with poorer survival endings in these patients. There have been meta-analysis studies on the impact of NLR and PLR on the survival and prognosis of lung carcinoma patients. Jin et al^[71]^ first analyzed 23 articles of 2068 patients with lung SCC treated with ICIs and then studied the correlation between NLR and the survival outcome of SCC patients. Li et al^[72]^ studied the correlation between NLR and those patients’ survival outcomes by summarizing 17 studies involving 2106 NSCLC patients treated with ICIs. Zhang et al^[32]^ analyzed 21 studies involving 1845 patients to study the survival outcome of NLR and PLR in those sufferers. The above researches have shown that high NLR and high PLR are related to the low survival rate of lung carcinoma patients treated with ICIs.

Compared with previous research, our research has the following advantages. Firstly, our analysis includes more projects than ever before. Before this meta-analysis, 2 studies evaluated NLR, and 1 study investigated NLR and PLR. The influence of LMR on the survival outcome of lung carcinoma patients treated with ICIs has not been explored. Compared with previous studies, we first comprehensively studied the prognostic survival effects of NLR, PLR, and LMR in NSCLC patients receiving ICIs. We discovered that all 3 markers are related to the prognosis of such patients. Secondly, the number of patients collected in our study is more significant than before. There are 34 studies in our research, including 3124 NSCLC patients receiving ICIs treatment, which is larger than the study population of Jing et al (2068), Li et al (2106), Zhang (1845), and others. To some extent, our results are more reliable than previous studies. Finally, since NSCLC is the primary category of lung carcinoma (around 80% of lung carcinoma cases), squamous cell carcinoma of the lung accounts for only 25%.^[4]^ Hence, we merely pay close attention to the survival outcome effects of NLR, PLR, and LMR on NSCLC, which will reduce the bias caused by different histological types. Unfortunately, the relevant literature has not distinguished between NLR, PLR, and LMR in patients with different subtypes of NSCLC treated with ICIs.

We should also admit that there are some shortcomings in this study. Firstly, all selected researches are retrospective English studies. Secondly, the cutoff value of NLR/PLR/LMR is different in the included studies. Various organizations use different standards and methods to determine the critical value, and we cannot put forward a suitable critical value through data analysis. This may lead to the inescapable potential heterogeneity of the results, thereby affecting the final results to influence the application of NLR/PLR/LMR in clinical work. Hence, it is necessary to define NLR/PLR/LMR standards and uniform cutoff values. Finally, other factors may influence the NLR/PLR/LMR value and survival outcome prognosis of patients, such as gender, age, smoking history, tumor malignancy, etc. Nevertheless, due to the lack of information provided by the original data, we can’t appraise the impact of distinct elements on the prognosis of NSCLC patients with NLR/PLR/LMR through stratified analysis.

Systemic inflammation is related to the prognosis of solid tumors. Because the complex interaction between T cells and other immune cells leads to the anticancer immune response, peripheral biomarkers are getting more and more attention, even in the context of immunotherapy. Among these hematological markers, NLR, PLR, LMR can reflect inflammation and host immune response. Higher NLR levels indicate increased inflammation of the original tumor and weak antitumor immunity.^[73]^ Based on the results of previous research, Tan et al^[74]^ proposed that the prognostic value of NLR in cancer patients treated with ICIs is related to the different functions of lymphocytes and neutrophils. Recruited neutrophils could stimulate the secretion of inflammatory cytokines, such as interleukin (IL)-1, IL-6, and tumor necrosis factor, and fuel a favorable environment for tumor development and progression.^[75]^ In contrast, lymphocytes are considered immune cells and exert antitumor effects. An increase in NLR means an increase in neutrophil count and/or a decrease in lymphocyte count; therefore, a higher NLR level reflects an antitumor superior to tumor-promoting activity, which means that patients receiving ICI treatment are an unfavorable prognostic factor. Moreover, low levels of circulating lymphocytes may react with lower levels of tumor-infiltrating lymphocytes and decreased antitumor T cells.^[76,77]^ These factors create an immunosuppressive tumor microenvironment that may reduce the likelihood of responding to ICIs. In addition, Parikh et al found possible differences in the cellular outcome of the interaction between monocytes and different tumor cell types. In response to certain environmental factors, such as activated lymphocytes, or under various pathophysiological conditions, monocytes undergo different phenotypic polarization into M1 or M2 macrophage subtypes.^[78]^ M1 macrophages are stimulated by cytokines such as interferon-γ, tumor necrosis factor, or Toll-like receptor ligands. They are characterized by enhanced antigen presentation, IL-12 and IL-23 production, and the ability to produce reactive oxygen species.^[79]^ M1 macrophages are cytotoxic, while M2 macrophages promote the growth of lung cancer xenografts.^[80]^ Therefore, due to the difference in the tumor microenvironment that regulates the polarization of tumor-associated macrophages, the effect of absolute monocyte count on ICIs response may be tumor type-specific. Studies have hypothesized that in patients treated with nivolumab, the increase in peripheral blood LMR may reflect the up-regulation of certain lymphocyte components or the down-regulation of certain monocyte components (such as myeloid-derived suppressor cells).^[81]^

## 5 Conclusion

The analysis shows that high pretreatment NLR and pretreatment PLR in NSCLC patients treated with ICIs correlate with low survival rates. Low LMR before and after treatment is also associated with a low survival rate. This suggests that NLR/PLR/LMR may be a useful prognostic indicator for patients receiving ICIs. It is necessary to use rigorously designed methods to conduct large-scale prospective studies to confirm our results.

## Author contributions

Hao Chi, Jinmei Mao, Wenhui Jia collected and analyzed the data. Chunling Dong acquired the funding. Na Liu and Peizhi Tao designed the study and wrote the first draft of the manuscript. Chunling Dong designed and supervised the study and finalized the manuscript, which all authors read and approved.

**Conceptualization:** Na Liu, Peizhi Tao, Hao Chi.

**Data curation:** Hao Chi, Jinmei Mao, Wenhui Jia.

**Formal analysis:** Na Liu, Hao Chi.

**Funding acquisition:** Chunling Dong.

**Methodology:** Na Liu, Peizhi Tao, Hao Chi, Jinmei Mao.

**Resources:** Chunling Dong.

**Software:** Na Liu, Peizhi Tao, Hao Chi.

**Supervision:** Chunling Dong.

**Validation:** Chunling Dong.

**Writing – original draft:** Na Liu, Peizhi Tao.

**Writing – review & editing:** Chunling Dong.

## Supplementary Material

Supplemental Digital Content

## Supplementary Material

Supplemental Digital Content
