# Supplementary material for: The relationship between NLR/PLR/LMR levels and survival prognosis in patients with non-small cell lung carcinoma treated with immune checkpoint inhibitors
Source: Medicine (Baltimore). 2022 Jan 21;101(3):e28617. doi: 10.1097/MD.0000000000028617 (PMC8772656; doi:10.1097/MD.0000000000028617)
Supplement: Supplemental Digital Content [file medi-101-e28617-s001.docx]

**EMBASE**

('nivolumab' OR 'pembrolizumab' OR 'atezolizumab' OR 'durvalumab' OR 'ctla-4' OR 'immune checkpoint inhibitor' OR 'pd-1' OR 'pd-l1') AND ('lung neoplasms' OR 'lung cancer' OR 'non-small cell lung cancer' OR 'nsclc') AND ('neutrophil to lymphocyte ratio' OR 'neutrophil lymphocyte ratio' OR 'neutrophil-to-lymphocyte ratio' OR 'neutrophil–lymphocyte ratio' OR 'neutrophil/lymphocyte ratio' OR 'nlr' OR 'platelet to lymphocyte ratio' OR 'platelet lymphocyte ratio' OR 'platelet–lymphocyte ratio' OR 'platelet-to-lymphocyte ratio' OR 'platelet/lymphocyte ratio' OR 'plr' OR 'lymphocyte monocyte ratio' OR 'lymphocyte–monocyte ratio' OR 'lymphocyte to monocyte ratio' OR 'lymphocyte-to-monocyte ratio' OR 'lymphocyte/monocyte ratio' OR 'lmr')

**PUBMED**

("Nivolumab"[All Fields] OR "Pembrolizumab"[All Fields] OR "Atezolizumab"[All Fields] OR "Durvalumab"[All Fields] OR "CTLA-4"[All Fields] OR "immune checkpoint inhibitor"[All Fields] OR "PD-1"[All Fields] OR "PD-L1"[All Fields]) AND ("lung neoplasms"[MESH] OR "lung cancer"[All Fields] OR "non-small cell lung cancer"[All Fields] OR "NSCLC"[All Fields]) AND (("neutrophil to lymphocyte ratio"[All Fields] OR "neutrophil lymphocyte ratio"[All Fields] OR "neutrophil-to-lymphocyte ratio"[All Fields] OR "neutrophil–lymphocyte ratio"[All Fields] OR "neutrophil/lymphocyte ratio"[All Fields] OR "NLR"[All Fields]) OR ("platelet to lymphocyte ratio"[All Fields] OR "platelet lymphocyte ratio"[All Fields] OR "platelet–lymphocyte ratio"[All Fields] OR "platelet-to-lymphocyte ratio"[All Fields] OR "platelet/lymphocyte ratio"[All Fields] OR "PLR"[All Fields]) OR ("lymphocyte monocyte ratio"[All Fields] OR "lymphocyte–monocyte ratio"[All Fields] OR "lymphocyte to monocyte ratio"[All Fields] OR "lymphocyte-to-monocyte ratio"[All Fields] OR "lymphocyte/monocyte ratio"[All Fields] OR "LMR"[All Fields]))

**Web of science**

# 1

67,890

TS=(Nivolumab OR Pembrolizumab OR Atezolizumab OR Durvalumab OR CTLA-4 OR immune checkpoint inhibitor OR PD-1 OR PD-L1)

# 2

588,205

TS=(lung neoplasms OR lung cancer OR non-small cell lung cancer OR NSCLC)

# 3

20,735

TS=(neutrophil to lymphocyte ratio OR neutrophil lymphocyte ratio OR neutrophil-to-lymphocyte ratio OR neutrophil–lymphocyte ratio OR neutrophil/lymphocyte ratio OR NLR)

# 4

7,572

TS=(platelet to lymphocyte ratio OR platelet lymphocyte ratio OR platelet–lymphocyte ratio OR platelet-to-lymphocyte ratio OR platelet/lymphocyte ratio OR PLR)

# 5

5,738

TS=(lymphocyte monocyte ratio OR lymphocyte–monocyte ratio OR lymphocyte to monocyte ratio OR lymphocyte-to-monocyte ratio OR lymphocyte/monocyte ratio OR LMR)

# 6

27,636

#5 OR #4 OR #3

# 7

214

#6 AND #2 AND #1

**Cochrane**

("Nivolumab" OR "Pembrolizumab" OR "Atezolizumab" OR "Durvalumab" OR "CTLA-4" OR "immune checkpoint inhibitor" OR "PD-1" OR "PD-L1") AND ("lung neoplasms" OR "lung cancer" OR "non-small cell lung cancer" OR "NSCLC") AND (("neutrophil to lymphocyte ratio" OR "neutrophil lymphocyte ratio" OR "NLR") OR ("platelet to lymphocyte ratio" OR "platelet lymphocyte ratio" OR "PLR") OR ("lymphocyte-to-monocyte ratio" OR "lymphocyte/monocyte ratio" OR "LMR")
