# Supplementary material for: The relationship between NLR/PLR/LMR levels and survival prognosis in patients with non-small cell lung carcinoma treated with immune checkpoint inhibitors
Source: Medicine (Baltimore). 2022 Jan 21;101(3):e28617. doi: 10.1097/MD.0000000000028617 (PMC8772656; doi:10.1097/MD.0000000000028617)
Supplement: Supplemental Digital Content [file medi-101-e28617-s002.docx]

**Supplementary Table A2. Quality assessment by the Newcastle-Ottawa Scale**

| **Authors** | **Year** | **Selection** | **Comparability** | **Outcomes** |
| --- | --- | --- | --- | --- |
|  |  |  |  |  |
| Bagley | 2017 | *** | * | ** |
| Diem | 2017 | *** | ** | ** |
| Patil | 2017 | ** | ** | ** |
| Rogado | 2017 | *** | * | ** |
| Soyano | 2017 | *** | ** | ** |
| Facchinetti | 2018 | *** | ** | *** |
| Fukui | 2018 | *** | ** | ** |
| Park | 2018 | *** | ** | ** |
| Russo | 2018 | *** | ** | ** |
| Shiroyama | 2018 | *** | ** | ** |
| Suh | 2018 | *** | * | *** |
| Takeda | 2018 | *** | ** | *** |
| Alona | 2018 | *** | ** | *** |
| Amaral | 2019 | *** | ** | ** |
| Dusselier | 2019 | *** | ** | *** |
| Katayama | 2019 | *** | ** | ** |
| Liu | 2019 | *** | ** | * |
| Miriam | 2019 | ** | ** | * |
| Ren | 2019 | *** | ** | *** |
| Pavan | 2019 | *** | ** | *** |
| Prelaj | 2019 | *** | ** | *** |
| Jiang | 2020 | *** | ** | ** |
| Katayama | 2020 | *** | ** | ** |
| Matsubara | 2020 | *** | * | ** |
| Prelaj | 2020 | *** | ** | *** |
| Petrova | 2020 | *** | ** | ** |
| Peng | 2020 | ** | ** | ** |
| Rossi | 2020 | *** | ** | ** |
| Russo | 2020 | *** | ** | ** |
| Simonaggio | 2020 | *** | ** | ** |
| Song | 2020 | *** | ** | * |
| Takada | 2020 | *** | ** | ** |
| Xiong | 2020 | *** | ** | * |
| Yuan | 2020 | *** | ** | ** |
